# Clinical and Demographic Characteristics of Patients with Urinary Tract Hydatid Disease

**DOI:** 10.1371/journal.pone.0047667

**Published:** 2012-11-02

**Authors:** Mou Huang, Hong Zheng

**Affiliations:** 1 Department of Urology, First Affiliated Hospital of Xin Jiang Medical University, Urumqi, XinJiang, China; 2 Department of Anesthesiology, First Affiliated Hospital of Xin Jiang Medical University, Urumqi, XinJiang, China; Queensland Institute of Medical Research, Australia

## Abstract

**Background:**

Human cystic echinococcosis (CE) is caused by flatworm larvae of *Echinococcus granulosus* and is endemic in many parts of the world. In humans, CE cysts primarily affect the liver and pulmonary system, but can also affect the renal system. However, the clinical manifestations of renal CE can be subtle, so healthcare professionals often overlook renal CE in differential diagnosis. In this study, we examined the clinical and demographic characteristics of patients with urinary tract CE and analyzed the diagnosis and treatment procedures for this disease.

**Methods:**

The records of 19 consecutive renal CE patients who were admitted to the First Affiliated Hospital of Xinjiang Medical University from January 1983 to April 2011 were retrospectively reviewed. In all cases, CE of the urinary tract was confirmed by pathological examination and visual inspection during surgery.

**Results:**

Fifteen patients were males and 4 were females. The most common symptoms were non-specific lower back pain and percussion tenderness on the kidney region. All patients were followed up for 9–180 months after surgery. None of the patients experienced a recurrence of renal CE, but 4 patients experienced non-renal recurrence of hydatid disease.

**Conclusions:**

Hydatid cysts from *E. granulosus* are structurally similar in the liver and urinary tract. Thus, the treatment regimen for liver CE developed by the World Health Organization/Informal Working Group on Echinococcosis (WHO/IWGE) could also be used for urinary tract CE. In our patients, the use of ultrasound, computed tomography, serology, and clinical characteristics provided a diagnostic accuracy of 66.7% to 92.3%.

## Introduction

Cystic echinococcosis (CE), also known as hydatid disease, is a condition caused by infection with the larval form (metacestode) of the parasitic tapeworm *Echinococcus granulosis*. CE is endemic in many parts of the world and is especially common in developing regions where stockbreeding is a major industry, such as Northwest China [1], [2], [3], [4]. Various herbivorous livestock species serve as intermediate hosts, dogs typically serve as definitive hosts, and humans may be considered as accidental hosts. Humans presumably acquire CE by ingestion of food contaminated with embryonated eggs of *E. granulosus* [3], [4], [5]. Once the eggs hatch in the intestines of the human host, the embryos (oncospheres) can enter the circulatory system, invade various organs, and develop into hydatid cysts that can cause life-threatening complications [5]. CE can affect all organs, but hepatic cysts account for about 75% [4], [5], [6], [7], pulmonary cysts for about 15%, and renal cysts for about 2–4% of cases. CE rarely occurs in the kidneys alone [4], [5], [6], [7].

Depending on the site of invasion, *Echinococcus granulosis* can cause cystic echinococcosis (CE) in various organs, including the urinary tract, liver, lung, or brain. The only definitive diagnostic sign of urinary tract CE is the presence of daughter vesicles in the urine, but this only occurs in 10–20% of patients with CE [8]. Infected patients may present without the typical clinical symptoms, imaging features [9], or serology, leading to a low reported incidence of urinary tract CE. The common symptoms of renal CE are generally non-specific and subtle and include the gradual development of waist/abdominal masses accompanied by lower back pain. These symptoms may have a slow onset and can be easily overlooked or disregarded by healthcare professionals, so there is a low rate of diagnosis before surgery [4].

Clinical trials of urinary tract CE are rare because the incidence of this disease is very low, often not allowing for a sufficient number of cases to carry out an analysis of treatment effectiveness stratified by cyst stage [10]. Although many cases of this disease have been reported in the literature, complete elucidation of pathophysiology of urinary tract CE is needed to develop accurate and rapid pre-surgical diagnoses for people who live in regions where this disease is common [11]. In the present study, we retrospectively analyzed the demographic characteristics and clinical features of patients from Northeastern China who presented with renal and urinary tract CE.

## Materials and Methods

### Patients

The records of 19 consecutive patients with urinary tract CE who were hospitalized at the First Affiliated Hospital of Xinjiang Medical University (Urumqi, Xinjiang) from January 1983 to April 2011 were retrospectively analyzed. Data was obtained from hospital records and records from the Department of Pathology. The demographic characteristics (age, gender, ethnic group, residence, history of exposure to sheep and dogs) and clinical characteristics (symptoms, physical signs, location of lesions, serology, imaging, treatment) of all patients were analyzed. All patients underwent routine preoperative examinations, and results in all cases indicated general anesthesia and surgery would be tolerable. This preoperative examination included routine blood and urine tests, liver and renal function tests, measurement of electrolytes, and the coagulation function test. This retrospective study was approved by the Institutional Review Board of First Affiliated Hospital of Xin Jiang Medical University and written consent was obtained.

### Diagnostic and Classification Criteria for Urinary CE

Urinary tract CE has similar structural features to the more common hepatic hydatid disease and can be characterized as caused by CL, CE1, CE2, CE3a, CE3b, CE4, or CE5 types, based on WHO/IWGE guidelines [12], [13], [14], [15], [16]. All patients underwent B-mode ultrasound or computed tomography (CT) scanning. Patients with suspected echinococcosis underwent serological examination. Our hospital stopped performing the Casoni skin test after the year 2000, at which time we adopted serological analysis to determine the presence of antibodies to serum echinococcus granulosus cyst fluid antigen B (EgB), and alveolar echinococcosis-specific antigen (Em2).

### Surgery

All patients were treated with various conservative or radical surgeries. Conservative surgery included simple internal endocyst excision plus drainage and internal capsule excision plus partial external pericyst wall resection [14], [15], [16], [17]. Radical surgery included pericystectomy for the renal hydatid cyst and partial nephrectomy [14], [18], [19]. No patients were treated with laparoscopic surgery or PAIR (puncture, aspiration, injection, and re-aspiration).

### Post Surgical Follow-up

Patient follow-up was performed for 9 to 180 months after surgery. Patients with early disease onset had longer follow-up periods. X-ray, B-mode ultrasound, or CT examination and serological analysis were performed during the follow-up examinations. X-ray analysis was used to detect the presence of hydatid cysts in the chest cavity, and B-mode ultrasound and CT scanning were used to detect the presence of a hydatid in the abdominal organs.

### Serological Analysis

The Rapid Diagnostic, Dot Immunogold Filtration Assay (DIGFA) Kit for Human Echinococcosis (Xinjiang Beisiming Biotechnology Development Co., Ltd.; Urumqi, Xinjiang) was used as previously described for the qualitative serological analysis of echinococcosis [20], [21]. Briefly, serum was extracted from all patient blood samples prior to the surgery. Two hydatid antigens (semi-purified EgB and Em2) were prepared by our institution and coated on the four corners of a nitrocellulose membrane. The membrane was dried at room temperature, and the test was performed on 13 out of the 19 samples, as specified by the manufacturer.

### Statistical Analysis

Continuous variables are expressed as medians and interquartile ranges (IQRs, 25^th^ and 75^th^ percentiles) and categorical variables as frequencies and percentages. The preoperative diagnostic accuracy of 3 methods were compared by Cochran’s Q test. All statistical analyses were performed with SAS software version 9.2 (SAS Institute Inc., Cary, NC), and two-tailed *p*-value less than 0.05 was considered statistically significant.

## Results

Table 1 shows the demographic and clinical characteristics of 19 patients who were admitted to our facility from January 1983 to April 2011 and were ultimately diagnosed with urinary tract hydatid disease. The 19 patients consisted of 15 males (79.0%) and 4 females (21.1%) and the median (IQR) age was 34 (23–48) years. More than half of the patients were of the Han ethnicity (52.6%), and most (79%) were employed. Only 3 patients (15.8%) reported a history of contact with dogs or sheep. Five patients had previous operations for CE and 3 patients had previous operations for kidney stones. The most common symptom was lower back pain (84.2%) and 2 subjects (10.5%) reported no symptoms. None of the patients experienced anaphylactic shock in response to treatment.

**Table 1:** Demographic and clinical characteristics of patients (n = 19) with urinary tract cystic echinococcosis.

Characteristic	
Age, median (IQR)	34 (23–48)
Gender, n (%)	
Male	15 (79.0)
Female	4 (21.1)
Ethnic group, n (%)	
Han	10 (52.6)
Uighur	6 (31.6)
Kazakh	3 (15.8)
Occupation, n (%)	
Unemployed†	4 (21.1)
Employed, not civil servant‡	6 (31.6)
Employed, civil servant	9 (47.4)
Contact with dogs or sheep, n (%)	
No	16 (84.2)
Yes	3 (15.8)
Surgery history, n (%)	
No	11 (57.9)
Hydatid disease	5 (26.3)
Kidney stone	3 (15.8)
Clinical symptoms§, n (%)	
Lower back pain	16 (84.2)
Upper abdominal pain	4 (21.1)
Fever	4 (21.1)
Percussion tenderness on the kidney	7 (36.8)
Other¶	4 (21.1)
None	2 (10.5)

† 3 students and 1 child,

‡ 3 workers and 3 farmers,

¶ 2 cases of waist and abdominal mass, 1 case of urine spine balloon, and 1 case of chest tightness,

§ Some patients had more than one clinical symptom.

Table 2 summarizes the clinical findings after surgery and follow-up. Twelve patients (63.2%) had lesions in the left kidney and 6 patients (31.6%) had lesions in the right kidney. Serological analysis was performed on samples from 13 of the 19 patients. We showed that 12 out of 13 patients (92.3%) were positive for the EgB antigen and none were positive for the Em2 antigen. After final pathological confirmation, 10 patients (52.6%) were classified as having *E. granulosus* renal disease only, and 8 patients (42.1%) with *E. granulosus* renal disease combined with hydatid disease in another organ(s). Seven patients (36.8%) suffered from a complication leading to leakage of urine. The median (IQR) duration of follow-up was 48 (16–110) months. At the last follow-up visit, none of the patients had evidence of renal CE recurrence, although 4 patients (19.1%) with combined CE of other organs had non-renal recurrence of hydatid disease.

**Table 2:** Summary of clinical findings after surgery and follow-up (n = 19).

Characteristic	
Location§, n (%)	
Left kidney	12 (63.2)
Right kidney	6 (31.6)
Liver	7 (36.8)
Others	5 (26.3)
Serology¶, n (%)	
EgB	12 (92.3)
Em2	0 (0.0)
Disease type†, n (%)	
* E. granulosus* renal disease	10 (52.6)
* E. granulosus* renal disease + hydatid disease in other organs	8 (42.1)
* E. granulosus* retroperitoneal disease	1 (5.3)
Complication, n (%)	
None	12 (63.2)
Leakage of urine	7 (36.8)
Follow-up duration, median (IQR)	48 (16–110)
Renal recurrence of hydatid disease, n (%)	
No	19 (100.0)
Yes	0 (0.0)
Non-renal recurrence of hydatid disease, n (%)	
No	15 (79.0)
Yes	4 (21.1)

§ Some patients had lesions in more than one location.

¶ Only 13 out of 19 patients underwent serological examination.

† Pathologically confirmed after operation. Four patients had extrarenal recurrence of hydatid disease.

**Figure 1:**
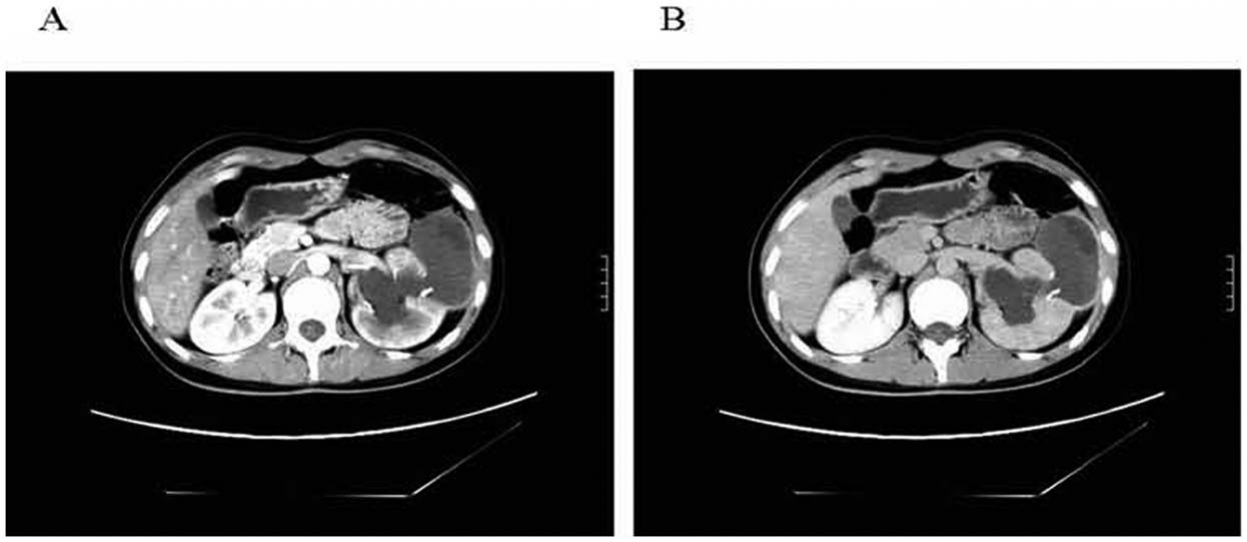
Type CE2 disease in a 17-year-old Uygur female patient. The patient experienced left lower back pain for three years and pain aggravation for two weeks with nausea. She had no history of exposure to sheep or dogs. Physical examination showed percussion tenderness over the left kidney region. A CT scan (A) showed connection of a CE cyst with the renal pelvis and an enhanced image indicated a thick-walled cystic lesion at the middle outer edge of the left kidney (6.7 cm×3.8 cm) (B). The boundary was clear, soft tissue septum, and multiple daughter vesicles and wall calcifications were also evident. The lesion communicated with the middle calyx and showed no enhancement. The left renal pelvis and calyx were dilated, and there was a patchy shadow of calcification.

**Figure 2:**
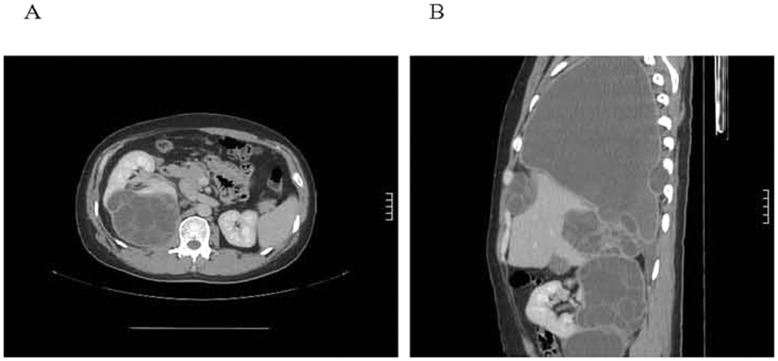
Type CE2 disease in a 35-year-old Han male patient. The patient experienced intermittent fever, shortness of breath, chest tightness, cough, and expectoration of a white-capsule-like substance for one month and aggravation of symptoms for 3 days. He did not experience any itching or palpitation. Physical examination showed dullness on percussion in the right lung and decreased respiratory movement. He underwent two surgeries for hepatic hydatid disease in 1995 and 2003. A CT image showed multiple hydatid cysts in the right lung (A), chest cavity (B), and diaphragm apex. Compression atelectasis of the right lung and a large amount of pleural effusion on the right side was observed. Multiple hepatic hydatid cysts and a renal hydatid cyst with multiple daughter vesicles were also observed.

Table 3 summarizes the classification of the imaging results based on the 2001 WHO/IWG-E classification of CE staging. Based on these guidelines, 10 patients (52.6%) had type CE2, 4 patients (21.1%) had type CE4, 2 patients (10.5%) had type CE5, and one patient each had types CL, CE1, and CE3a. All 10 patients with multivesicular hydatid cysts (CE2) had CE-specific signs (Figures 1 and 2). The CT images of all patients had only pericystic wall enhancement and no intracapsular enhancement.

**Table 3:** Classification of imaging results based on the WHO/IWG-E classification of cystic echinococcosis (n = 19).

WHO/IWG-Eclassification	Image characteristics	No (%)
CL	Univesicular, cystic lesion with uniform echoes, clear boundary, thin visible wall. If it is a hydatid cyst, it is active.	1 (5.3)
CE1	Univesicular anechoic cyst. Presence of hydatid sand, snow flake sign and double wall sign. The hydatid is active.	1 (5.3)
CE2	Multivesicular, multiseptated cysts; cyst septations produce “wheel-like” sign, and presence of daughter vesiclesis indicated by “rosette-like” or “honeycomb-like” structures. The hydatid is active.	10 (52.6)
CE3a	Detachment of laminated membrane from the cyst wall visible as “big snake sign” or as “water-lily sign”.The hydatid status is transitional.	1 (5.3)
CE3b	Intracystic shadow of the daughter vesicles and solid septation, manifested as complex cyst shadow.The hydatid is dying.	0 (0.0)
CE4	Heterogenous hypoechoic or hyperechoic contents in the cyst. No daughter vesicles.The hydatid is inactive.	4 (21.1)
CE5	Intracystic solid degeneration and calcification of the cystic wall. The hydatid is inactive.	2 (10.5)

All 19 patients underwent surgery, and postoperative pathology confirmed the presence renal CE disease. Six patients undergoing simple internal capsule excision experienced postoperative leakage of urine, which healed spontaneously after 3 months of double J catheter drainage or perirenal drainage. One patient underwent pericystectomy, and postoperative urine leakage occurred, which healed spontaneously after 3 months.

There were 4 types of operations given to the 19 CE patients (Table 4), and cystectomy was the most common procedure (15/19). Patients with renal CE and CE in other organs underwent transabdominal surgery, at which time intra-abdominal lesions were also treated. One case presented with CE in the chest cavity, lung, diaphragm, and liver. The CE in the thoracic cavity was treated first, and abdominal surgery was the secondary operation. Internal capsule excision was adopted for all the cysts. Four patients with combined CE of other organs experienced postoperative extrarenal recurrence.

**Table 4:** Types of surgery, presence of complications, and recurrence of non-renal hydatid disease (n = 19).

Type of Surgery	Total No.	Complication† (%)	Non-renal recurrence‡ (%)
Cystectomy	15	6 (40.0)	4 (26.7)
Cystectomy + Partial pericystectomy	1	0 (0.0)	0 (0.0)
Pericystectomy	1	1 (100.0)	0 (0.0)
Partial nephrectomy	2	0 (0.0)	0 (0.0)

† Only leakage of urine,

‡ 3 cases of recurrence in the liver and 1 case of recurrence in the lung.

The pre-operative diagnostic accuracy rate was 66.7% for ultrasound, 88.2% for CT, and 92.3% for serology when final pathological examination was used as the gold standard for diagnosis (Table 5). These differences were not significant (*p* = 0.223). Correct diagnosis was achieved in 14 of 19 patients before surgery, and the remaining 5 patients were diagnosed during surgery or by postoperative pathological results (Table 5). Ten of the 19 patients had imaging results typical of hydatid disease stage CE2 and this diagnosis was confirmed during or after surgery. One patient passed hydatid cysts in the urine, confirming the presence of renal CE. Five patients had previous histories of surgery due to CE, which provided diagnostic clues. Two patients who had undergone ipsilateral kidney stone surgery experienced lower back pain and underwent further B-mode ultrasound or CT examination; one of these patients was initially misdiagnosed as having cysts and infection, and the other as having stones and hydrocele. Three patients with lower back pain underwent CT scans and were initially misdiagnosed as having suspected cystic carcinoma.

**Table 5:** Pre-operative diagnostic accuracy of hydatid disease based on different methods (n = 19).

DiagnosticMethod	Total No.	Correctly diagnosedcases, No (%)	*p* †
Ultrasound	18	12 (66.7)	0.223
Computedtomography	17	15 (88.2)	
Serology	13	12 (92.3)	
Clinical	19	14 (73.7)	

† Cochran’s Q test was used to compare the pre-operative diagnostic accuracy rate of ultrasound, computed tomography, and serology.

## Discussion

Urinary tract CE can be difficult to diagnose due to the slow growth of cysts, the presence of non-specific and subtle clinical manifestations, and the rarity of daughter vesicles in the urine, the defining sign of this disease [4], [22], [23]. In this study of 19 patients with renal or urinary tract CE, 16 patients exhibited lower back pain and upper abdominal pain, 7 patients experienced percussion tenderness over the kidney region, but only 1 patient had daughter vesicles in the urine. Lower back pain and kidney tenderness are the most common symptoms of urinary tract CE, but these symptoms are clearly not specific, so urinary tract CE is often overlooked in differential diagnoses. Although urinary tract CE has a relatively low incidence, misdiagnosis can have major health consequences.

Our results indicate that pre-operative serological analysis of EgB and Em2 antibodies using the Rapid Diagnostic Kit for Human Echinococcosis had a 92.3% positive rate for detection of CE, comparable to other common serological detection methods such as ELISA [12], [20], [21], [23]. In this study, the diagnostic accuracy of serological analysis (92.3%) was higher than that from B-mode ultrasound (66.7%) or CT scans (88.2%), although these differences were not significant. In general, imaging by CT or ultrasound is considered the main tools for diagnosis, and serology and other tests are considered complementary [10]. Our results emphasize that serological analysis is an important pre-operative technique for the diagnosis of renal and urinary tract CE, especially in patients who exhibit no specific clinical symptoms or who have atypical CT or ultrasound results.

The WHO/IWG-E classification system for CE diagnosis and treatment is based on B-mode ultrasound medical imagery and classifies hydatid cysts as stage CL, CE1, CE2, CE3a, CE3b, CE4, or CE5 (Table 3) [10], [11]. CE1 and CE2 cysts are active and fertile with viable protoscoleces; CE3a and CE3b cysts are in a transitional stage where the integrity of the cyst has been compromised; and CE4 and CE5 cysts are inactive and degenerative [13]. Urinary tract CE is structurally similar to hepatic CE, so this classification system can also be used for the diagnosis of urinary tract CE. Thus, renal CE should be strongly suspected when a patient presents with renal ultrasound or CT images suggestive of a hydatid cyst, positive serological tests, and/or a history of residence in endemic areas [9], [14], [24].

B-mode ultrasound is accepted as the initial diagnostic tool for CE [10]. However, we found ultrasound and CT had similar diagnostic accuracy. The advantages of ultrasound are that it is simple, inexpensive, and provides instant results. However, CT scanning also has several advantages for the diagnosis of urinary tract CE [14], [25] in that it provides visualization of cyst wall calcification and intracystic infection, and (in contrast enhanced mode) also allows identification of low-density and high-density areas, which is important for the differentiation of a CE4 cyst from a urinary tract abscess or tumor.

Surgery is the main treatment option for urinary tract hydatid disease. Chemotherapy with or without puncture-aspiration-injection-re-aspiration (PAIR) may be performed in patients with inoperable disease [26], [27], [28], but none of our patients were treated by this methodology. In addition to being used on patients with type CE1 hydatid cyst, PAIR is also used to treat patients with hydatid disease containing several daughter cyst, who cannot tolerate surgery or who do not wish to undergo surgery. The goal of surgery is to remove all of the cyst contents and the pericyst in order to prevent spread and recurrence, while preserving as much functional kidney tissue as possible. Conservative surgery tends to have greater kidney preserving capabilities, and is appropriate for ∼75% of renal CE cases, but radical surgery is necessary for ∼25% of cases [13], [14], [15], [16], [17]. The conservative surgery, cystectomy with simple drainage [9], [21], [22], [23] was performed on 15 of 19 patients in this study. Postoperative urine leakage occurred in 6 of these cases, but no fistula was found in 5 cases before or after surgery. Urine leakage, which is assessed via a drainage catheter, healed spontaneously 3 months after placement of a double J catheter or drainage through the perirenal drainage tube. One patient experienced passage of a daughter vesicle in the urine, and a 0.5 cm fistula at the bottom of the cyst (linking with the renal pelvis) was found during surgery. Although the mechanism of urinary fistula formation remains unclear, it is possibly related to the hydatid cyst compressing the collection system. Some of our patients had hydatiduria, while some showed no significant clinical manifestations in spite of the presence of a urinary fistula. It is possible that the fistula existed before surgery and remained undetected during the surgery.

Urine leakage may affect postoperative rehabilitation so prevention of this is a major concern during simple cystectomy. In our study, 7 patients had urine leakage that did not heal for three months, so we suggest that the overall surgical approach should be improved to reduce the chance of this complication.

None of the patients in our study were given pre- or post-surgical albendazole. Administration of this agent for one week to one month before surgery may reduce the intraoperative tension of the CE cyst, prevent CE spread during puncture, and may kill or reduce the activity of *Echinococcus* larvae [10]. Continuous use of albendazole for 3 months after surgery may also reduce postoperative recurrence, especially when cystic fluid has spread during surgery [29], [30]. Use of this medication in our study could have potentially improved patient surgical outcomes.

In conclusion, a patient with lower back pain, upper abdominal pain, and/or percussion tenderness over kidney region, who lives in endemic areas and has a history of exposure to sheep or dogs, should undergo B-mode ultrasound, CT, and/or serological analysis for investigation of possible CE. If a complicated thick-walled urinary tract cyst is found, or serological analysis is positive, CE should be considered and further examination and treatment is necessary.
